# Potential FSH-mediated molecular pathway to regulate follicle development in striped hamsters (*Cricetulus barabensis*) supported by strong correlative evidence

**DOI:** 10.1371/journal.pone.0339880

**Published:** 2025-12-29

**Authors:** Huiliang Xue, Yunjiao Zheng, Chao Fan, Jinhui Xu, Lei Chen, Ming Wu, Laixiang Xu

**Affiliations:** College of Life Sciences, Qufu Normal University, Qufu, Shandong, People’s Republic of China; Al-Azhar University Faculty of Science for Boys in Cairo, EGYPT

## Abstract

**Scientific background:**

Rational control of rodent populations is crucial for maintaining ecosystem balance and mitigating agricultural economic losses. Follicle development plays a pivotal role in determining animal population abundance, and photoperiod serves as the primary environmental cue affecting this process. Investigating the mechanisms through which photoperiod influences follicle development in the striped hamster (*Cricetulus barabensis*) offers a promising molecular target for the effective and sustainable management of rodent populations.

**Methodology:**

This study employed hematoxylin and eosin (HE) staining to evaluate ovarian developmental status under different photoperiods, including quantification of follicles at various developmental stages and the number and thickness of granulosa cell layer, thereby elucidating the effects of photoperiod on follicle development. Subsequently, enzyme-linked immunosorbent assay (ELISA) was used to measure serum FSH and fecal E2 concentrations, while real-time quantitative PCR was performed to determine mRNA levels of *CCND1* and *CCND2*. Correlation analyses between these markers and follicle counts were conducted to identify key factors involved in follicle development. Furthermore, both real-time quantitative PCR and Western blotting were utilized to investigate the expression of transcription factors FOXO1, FOXL2, and NR5A2 in the ovary at the mRNA and protein levels, respectively, and their relationships with follicle numbers were analyzed, to reveal the potential molecular pathways through which photoperiod regulates follicle development in the striped hamster.

**Results:**

The results demonstrate that LP enhances the synthesis of FSH, promotes granulosa cell proliferation, and stimulates follicle development, whereas SP exerts an opposing effect in the striped hamster. FSH is a key hormone involved in follicle development regulated by photoperiods, and CCND2 influences follicle development by modulating granulosa cell proliferation. Additionally, photoperiod alters the expression levels of transcription factors FOXO1, FOXL2, and NR5A2. Correlation analyses revealed that serum FSH concentration was significantly positively correlated with the expression levels of FOXO1 and FOXL2. In turn, the expression of FOXO1 and FOXL2 was significantly positively associated with that of NR5A2, which also showed a significant positive correlation with CCND2 expression. These results suggest a potential regulatory pathway—FSH-FOX-NR5A2-CCND2—involved in photoperiod-dependent follicle development in the striped hamster.

**Conclusion:**

The FSH-FOX-NR5A2-CCND2 pathway represents a potential molecular mechanism by which photoperiod regulates follicle development, supported by robust correlative evidence in the striped hamster. The transcription factors FOXO1, FOXL2, and NR5A2 are identified as candidate targets of reproductive activity, with NR5A2 showing a stronger correlation than FOXO1 and FOXL2, thus providing a theoretical foundation for the rational control of rodent population dynamics.

## 1. Introduction

A demographically sustainable animal population is critical for preserving ecological equilibrium. Seasonal reproduction constitutes a critical factor influencing temporal dynamics in animal population fluctuations. Animals exhibiting seasonal reproductive patterns modulate their reproductive behaviors in response to photoperiodic cues [1], thereby ensuring that offspring are born during periods of maximal survival suitability [2]. Various species demonstrate distinct physiological and behavioral mechanisms in response to photoperiodic regulation of reproductive activities. Some species reproduce during extended photoperiods [3], whereas others initiate breeding under shortened photoperiods [4].

Photoperiod primarily regulates seasonal reproductive activities in animals via the hypothalamic-pituitary-gonadal axis (HPGA) [5,6]. In seasonal breeding animals, light signals are initially converted into neural impulses by retinal ganglion cells. These impulses are then transmitted to the pineal gland via the suprachiasmatic nucleus (SCN), where they are translated into endocrine signals [7]. The pineal gland subsequently synthesizes and secretes melatonin (MT). Melatonin interacts with its receptors located on the gonadotropin-releasing hormone (GnRH) neurons in the hypothalamus, thereby modulating the synthesis and secretion of GnRH [8]. GnRH further governs the synthesis and secretion of luteinizing hormone (LH) and follicle-stimulating hormone (FSH) in the anterior pituitary gland. Thereafter, these gonadotropins—LH and FSH—are transported via the bloodstream to the gonads, where they stimulate gonadal development and consequently regulate the reproductive activities of animals [9,10]. The precise mechanisms and signaling pathways by which gonadotropins exert their effects on the gonads remain to be elucidated.

In female animals, follicle development serves as a critical and highly regulated event in the reproductive process. Investigating the molecular mechanisms of follicle development responding to photoperiods facilitates the rational control of the animal reproductive activity and, consequently, the regulation of animal population size for maintaining ecological balance.

Analyzing the key regulatory factors involved in the signaling pathways that govern follicle development, and based on these insights, designing specific pharmacological agents capable of modulating follicle development in animals, offer a scientifically sound strategy for rationally managing animal population sizes and contribute to the maintenance of ecological balance. Compared with traditional rodent control methods that rely on chemical agents, the proposed approach exhibits higher specificity, poses reduced risks to non-target species, minimizes environmental pollution, and represents a safer and more eco-friendly strategy for rodent management.

The ovaries of rodents harbor a substantial number of primordial follicles, each consisting of an early-stage oocyte enveloped by a layer of flat granulosa cells [11,12] and located in the outer cortical region of the ovary [13,14]. After formation, primordial follicles enter a state of quiescence and dormancy, collectively constituting the dormant pool [15,16]. Upon leaving the dormant pool and being recruited into the growing follicle pool, primordial follicles undergo a transition wherein granulosa cells change from a flat morphology to a cuboidal morphology and begin to express cell proliferation markers [17], thereby developing into primary follicles. In the primary follicle, the granulosa cell layers transition from a single layer to multiple layers, concurrent with an increase in oocyte volume [18]. This process signifies the development of the primary follicle into a secondary follicle, during which a complete follicle membrane and zona pellucida are formed. As secondary follicles continue to develop, spaces containing follicular fluid begin to form between granulosa cells. These spaces eventually coalesce into a single cavity, known as the follicular antrum or follicular cavity [19], marking the transition of secondary follicles into antral follicles. Concurrently, the oocyte and its surrounding granulosa cells form the cumulus oophorus. The majority of follicles undergo atresia, while only a small fraction progress to the pre-ovulatory stage [20]. As the follicle cavity progressively enlarges, the cumulus oophorus matures and releases the oocyte during ovulation. Following ovulation, the follicle wall collapses inwardly, accompanied by capillary hemorrhage, leading to the formation of luteal cells [21]. Therefore, the development status of follicles directly affects the intensity of reproductive activities in animals.

The follicle is primarily composed of oocytes, granulosa cells (GCs), and theca cells, each playing a distinct role in follicle development and function [22]. Granulosa cells play a critical role in regulating the synthesis and secretion of growth factors associated with oocyte maturation [23,24], and follicle development is significantly dependent on the proliferation of these cells [25]. Studies have demonstrated that when the proliferation of granulosa cells in rat follicles is compromised, the thickness of the granulosa cell layer diminishes, the number of secondary follicles and antral follicles decreases, and the development of rat follicles is significantly impaired [26]. Therefore, granulosa cells play a pivotal role in the development of animal follicles, and their proliferation directly influences the developmental status of the follicles. Cyclin D1 (CCND1) and Cyclin D2 (CCND2) are members of the highly conserved D-type cyclin family. By activating cyclin-dependent kinases CDK4 or CDK6, CCND1 and CCND2 drive the progression of cells from the G1 phase to the S phase, thereby promoting cell proliferation [27]. CCND1 and CCND2 are essential regulatory factors for the proliferation of granulosa cells and the development of follicles in mammals [28,29].

The nuclear receptor superfamily is a distinct class of transcription factors that plays an essential role in regulating cellular signal transduction and proliferation processes [30,31]. Among these, NR5A2 (Nuclear Receptor Subfamily 5, Group A, Member 2), also referred to as Liver Receptor Homolog-1 (LRH-1), serves as one of the critical transcription factors that regulate granulosa cell proliferation and follicle development. Studies have demonstrated that NR5A2 plays a critical role in regulating the expression of genes associated with embryonic development, metabolic regulation, steroid hormone synthesis, follicle maturation, and ovulation [32]. Some studies also have demonstrated that NR5A2^-/-^ mouse embryos are nonviable, whereas fertility is reduced in NR5A2^+/-^ female mice [33], indicating that NR5A2 plays a critical role in regulating reproductive functions in female mammals. In addition, studies have demonstrated that the promoter region of *NR5A2* is enriched in specific binding sites for the Forkhead box (FOX) transcription factors, and FOX regulates *NR5A2* expression through specific binding to its promoter [34].

Members of the FOX transcription factor family play essential roles in regulating animal growth, development, and metabolic processes [35]. Notably, Forkhead box L2 (FOXL2) is predominantly expressed in the ovary, eyelid, and pituitary gland, where it critically contributes to gonadal differentiation, follicle maturation, and estradiol biosynthesis [36]. Studies have demonstrated that FOXL2^-/-^ female mice exhibit impaired follicle development, ovarian dysgenesis, and significantly reduced reproductive capacity [37]. FOXL2 is predominantly expressed in the granulosa cells of ovarian follicles. Knocking out the FOXL2 gene in granulosa cells using CRISPR technology suppresses the expression of genes associated with cell proliferation and inhibits follicle development [38,39]. Some studies have further demonstrated that Forkhead box O1 (FOXO1) is a critical transcription factor involved in the regulation of follicle development [40]. FOXO1 is predominantly expressed in the granulosa cells of mammalian ovaries and regulates the expression of genes associated with cell proliferation [41]. Knockout of the FOXO1 gene in granulosa cells significantly decreases their proliferative capacity, impairs follicle development, and ultimately results in infertility in mice [42]. Therefore, the transcription factors FOXL2, FOXO1, and NR5A2 play crucial roles in regulating granulosa cell proliferation, which in turn affects animal follicle development [43].

Upon binding of FSH to its receptor FSHR, it triggers intracellular signal that activates serine/threonine kinases in the PI3K/Akt or LATS1 pathways. This activation modulates the activity of FOX family transcription factors, thereby regulating the expression of genes associated with granulosa cell proliferation and follicle development [44–46]. Investigating the biological roles of transcription factors FOXL2, FOXO1, and NR5A2 in regulating granulosa cell proliferation and follicle development via the FSH signaling pathway contributes to the rational management of animal reproductive activity levels.

The striped hamster is one of the primary agricultural pests in North China, exhibiting typical seasonal reproductive traits. Photoperiod serves as the key environmental factor influencing its seasonal reproductive activities. However, the mechanisms and effects of transcription factors FOXL2, FOXO1, and NR5A2 in mediating photoperiodic regulation of follicle development in striped hamsters remain unclear. This study employed adult female striped hamsters as experimental subjects to investigate the biological roles of transcription factors FOXL2, FOXO1, and NR5A2 in regulating follicle development under varying photoperiod conditions. The findings provide a theoretical foundation for the rational management of farmland pest populations and the preservation of ecological balance.

## 2. Materials and methods

### 2.1. Sample collection and photoperiod treatments

The striped hamsters were captured using the live-trap method in the field from Wu Village (116°59′E, 35°58′N), Qufu City, Shandong Province, China. The sampling site for this study is situated in an open area within mountainous and hilly regions, where some plots have been reclaimed by local farmers, while others belong to collectively managed land that has been contracted to them. Capturing rodents without disturbing the crops helps minimize agricultural losses caused by rodent infestations and is therefore strongly supported by local farmers cultivating the land. The captured hamsters were identified, individually numbered, and housed in a feeding room with natural lighting. They were provided with composite rat food pellets, which were purchased from Jinan Peng Yue Experimental Animal Breeding co.,Ltd., and applied with water ad libitum. The composite rat food pellets are formulated to meet the specifications of standard laboratory rodent chow, with a nutrient profile comprising ≥220 g/kg crude protein, ≥ 80 g/kg crude fat, ≤ 50 g/kg crude fiber, and ≤80 g/kg crude ash, along with 10–16 g/kg calcium and 6–10 g/kg phosphorus. This composition ensures full compliance with the nutritional requirements of laboratory hamsters. All experimental procedures were reviewed and approved by the Biomedical Ethics Committee of Qufu Normal University (Permit Number: 20190307) and conducted in accordance with the guidelines set by the Chinese Experimental Animal Ethics Committee.

Eighteen adult female striped hamsters (about 8 months of age) were selected based on the estimation of molar wear, a common indicator used to determine the age of mammals, particularly rodents [47] and its body weight was measured. The selected individuals were randomly divided into two groups, each consisting of 9 female hamsters. Two distinct photoperiods were applied: a short photoperiod (light: darkness = 8 h:16 h; SP), simulating a non-breeding season, and a long photoperiod (light: darkness = 16 h:8 h; LP), mimicking a breeding season. Subsequently, the two groups were maintained separately under SP and LP conditions at 22 ± 2°C, 55% ± 5% RH, and a light intensity of 150 ± 10 lx for 8 weeks.

### 2.2. Sample preparation

Following an 8-week photoperiod exposure, the striped hamsters were humanely euthanized with carbon dioxide. They were immediately weighed and dissected for sample collection. The fallopian tubes and fat surrounding the ovaries were carefully removed, and the weight of both ovaries was recorded. The ovarian coefficient was then calculated (ovarian coefficient = wet weight of both ovaries ÷ body weight × 100%). The left ovary was fixed in 4% paraformaldehyde for subsequent paraffin embedding and hematoxylin and eosin (H&E) staining. The remaining ovaries were stored at −80 °C for subsequent qPCR and Western blot analyses. The striped hamsters’ serum and feces were collected for hormone concentration analysis.

### 2.3. Histological study

After the ovaries were weighed, they were immediately fixed in 4% paraformaldehyde solution for 24–48 hours. Subsequently, the ovarian tissues were processed through dehydration, wax infiltration, embedding, sectioning, and mounting. The resulting paraffin-embedded sections were dewaxed, stained with hematoxylin-eosin, dehydrated, and mounted for microscopic examination and image acquisition. For sectioning, continuous slices of approximately 5 µm in thickness were prepared. One section was selected from the middle region of the ovary, and one additional section each from the upstream and downstream directions, spaced at intervals of four consecutive sections. A total of three representative sections per ovary were examined to quantify follicle numbers and assess the number of granulosa cell layers. Three fields of view were examined per section. The selection of fields and the enumeration of follicles within each field were performed systematically from left to right and from top to bottom to avoid duplicate or missed counts, thereby ensuring the accuracy and consistency of follicle quantification. The total number of follicles across three fields of view on each section was used as a single follicle count parameter.

Follicles at different developmental stages were defined as follows. Primary follicles are characterized by a single layer of cuboidal granulosa cells. Secondary follicles exhibit multiple layers of granulosa cells and a fully formed zona pellucida. Antral follicles are defined by the presence of a well-defined follicular antrum along with multiple layers of granulosa cells. In luteinized follicles, HE staining displays reduced intensity, and the granulosa cells differentiate into larger, luteinized granulosa cells. These analyses allowed for the observation of differences in ovarian morphology and structure, as well as the quantification of follicles at different developmental stages between the LP and SP groups. Additionally, the number of layers and thickness of granulosa cells in primary follicles, secondary follicles, and antral follicles were statistically analyzed.

### 2.4. Hormone concentration detection

After the completion of the photoperiod treatment, fecal samples were collected from female striped hamsters. According to the ratio of 5 mL of physiological saline per 1 g of feces, an appropriate amount of physiological saline was added to the feces, which were thoroughly homogenized and then centrifuged. The resulting supernatant was transferred to sterilized centrifuge tubes and stored at −80°C. Following euthanasia, blood was collected into sterilized centrifuge tubes. The tubes were incubated at 4°C until initial layer separation occurred, after which they were immediately centrifuged using a high-speed refrigerated centrifuge. After centrifugation, the supernatant was transferred to sterilized centrifuge tubes and stored at −80°C. The E2 content in the fecal samples and the serum FSH concentration was determined using Enzyme-Linked Immunosorbent Assay (ELISA) method according to the kit instructions (Shanghai Jining Biotechnology Co., Ltd., JN82367, JN83272, Shanghai, China).

### 2.5. Quantitative real-time PCR

Total RNA from the ovaries was extracted using TRIzol reagent (TaKaRa, Dalian, China) according to the manufacturer’s instructions [48]. The quality of the extracted RNA was assessed based on the A260/A280 ratio, while its integrity was evaluated using agarose gel electrophoresis (AGE). Using the TaKaRa reagent, the qualified RNA was reverse-transcribed into cDNA and stored at −80°C for subsequent using. The experiment was performed using the SYBR® Green Premix HS qPCR Kit II (Accurate Biotechnology Co., Ltd., Changsha, Hunan, China). The amplification efficiency of the gene-specific primers ranged from 90% to 110%, and the coefficient of determination (R²) was greater than 0.99. To minimize the error, each gene was assayed in triplicate, and the difference in threshold cycle (Ct) values among replicates was less than 0.5. The mRNA expression levels of the target genes, including *CCND1*, *CCND2*, *FOXO1*, *FOXL2*, and *NR5A2*, were normalized to that of the housekeeping gene (β-actin) using the 2^-ΔΔCT^ method [49]. The differences in the expression levels of the same gene among individuals from different photoperiods were analyzed using a one-way analysis of variance (ANOVA) followed by Fisher’s least significant difference (LSD) testing. The specific information of all primers is detailed in Table 1.

**Table 1:** Primers of quantitative real-time fluorescent PCR.

Primer	Primer sequences (5′ → 3′)	Product length (bp)	Annealing temperature (°C)
*FOXL2*	F: CCGGGATGCCAGGAGATTAC	124	60
	R: GGCCGGTTTCACATTTCTCC		
*FOXO1*	F: CCCACCCTGGACATTCACAA	108	57
	R: TCATGGGAGTCAAGCGGTTC		
*NR5A2*	F: CCCAGCCAGCATCCCACATC	140	60
	R: GCCCAAACGTGTTCAGCTTTCC		
*CCND1*	F: CAAGTGTGACCCGGACTGC	100	60
	R: GGCCTTGGGGTTGATGTTCT		
*CCND2*	F: TGTGTTGAGGTGTGCAGTGT	86	61
	R: GGGTATCGGCCACCAAAGAA		
*β-actin*	F: GAGACCTTCAACACCCCAGC	256	n
	R: ATGTCACGCACGATTTCCC		

### 2.6. Western blotting

#### 2.6.1. Extraction of total protein from ovarian tissue.

The total protein of the ovaries was extracted using RIPA lysis buffer (G2002-100ML, Servicebio, Wuhan, China), a protease inhibitor cocktail (G2007-1ML, Servicebio, Wuhan, China), and PMSF (G2008-1ML, Servicebio, Wuhan, China). Ovaries were retrieved from the −80 °C freezer, minced, and thoroughly homogenized after adding RIPA lysis buffer, PMSF, and phosphatase inhibitor cocktail. The mixture was vortexed every 10 minutes for a total of three times and then incubated on ice for 30 minutes. Subsequently, the samples were centrifuged at 12,000 × g for 15 minutes at 4 °C, and the supernatant was collected. The volume of the supernatant was recorded, and 6 μl total protein was used to determine the protein concentration using the BCA Protein Assay Kit (AR1189, BOATER).

#### 2.6.2. Protein concentration determination.

The standard curve was firstly constructed based on the BSA standards prepared according to the kit instructions. Then, the protein concentration of the samples was measured using the microplate method. Finally, the protein concentration was calculated by incorporating the absorbance values obtained at 562 nm into the established standard curve.

#### 2.6.3. Separating proteins by SDS-PAGE gel electrophoresis.

First, the clean glass plates were carefully assembled, and the separating gel was evenly applied to the gap between the glass plates. After the separation gel had solidified, the comb was inserted, and the concentrated gel was added. After the concentrated gel had solidified, the gel plate was transferred to the electrophoresis tank, the electrophoresis buffer was added, and then the comb was removed. The protein samples were added to the gel wells, and electrophoresis was performed at 80 V until the bromophenol blue reached the bottom of the glass plate. The separation gel was then removed and placed on the gel imaging system to observe the protein bands.

#### 2.6.4. Blotting transfer.

The foam board, thick filter paper, and methanol-activated PVDF membrane were pre-soaked in the transfer buffer solution. Then, the foam board, thick filter paper board, PVDF membrane, gel, thick filter paper board and foam board were placed in the transfer sandwich in order, and any air bubbles were removed using a glass rod. Lastly, the assembly was transferred into the transfer tank and electrophoretically transferred at a constant current of 200 mA for 140–150 minutes.

#### 2.6.5. Sealling the membrane.

After the transfer process was completed, the PVDF membrane was washed once with TBST buffer to remove any non-specifically bound substances. Based on the position of the molecular weight marker, the membrane region containing the target protein was excised and transferred to a blocking solution prepared with 5% skim milk in Tris-buffered saline with Tween 20 (TBST). The membrane was then incubated at 37°C on a shaker for 1 hour to block non-specific binding sites.

#### 2.6.6. Protein immune response.

The sealed PVDF membrane was rinsed with TBST buffer, transferred to an incubation bag containing the diluted primary antibody solution, and incubated at 4°C for 13−14 hours. The catalog numbers for the antibodies against FOXO1, FOXL2, and NR5A2 are 18592-1-AP, 19672-1-AP, and 22460-1-AP, respectively, with corresponding dilutions of 1:4000, 1:2000, and 1:1500. After the incubation, the membrane was washed four times with TBST buffer on a shaker at room temperature for 15 minutes per wash. The membrane was transferred to the incubation box containing the diluted secondary antibody and incubated at 37 °C for 2 hours. After the incubation, the membrane was washed six times with TBST buffer for 15 minutes per wash at room temperature.

#### 2.6.7. Protein band staining.

After the PVDF membrane was incubated with the luminescent liquid for approximately 20–30 seconds, it was exposed to visualization in the gel imaging instrument. The image was saved, and the optical density of the target protein was analyzed using Image J software. Subsequently, the protein expression level was normalized against the internal reference protein.

### 2.7. Statistical analyses

Experimental data were presented as mean ± standard deviation. Normality (Shapiro-Wilk) and homogeneity of variance (Levene) tests were conducted using IBM SPSS Statistics 27. The independent samples t-test was utilized to evaluate intergroup differences, whereas GraphPad Prism 5 software was employed to perform the correlation analysis of the data. The correlation analyses were performed using individual data points from animals in Fig 4, Fig 5 and in Table 1.

## 3. Results

### 3.1. Differences in the body weight, ovary weight and ovary coefficient of the striped hamsters (*Cricetulus barabensis*) under different photoperiods

**Fig 1:**
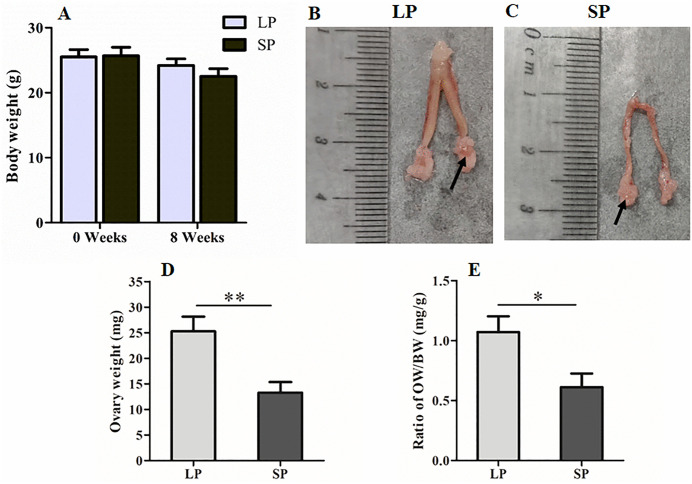
Comparison of body weight, ovarian weight, and ovarian coefficient in the striped hamsters exposed to different photoperiods. A, Body weight; B, Reproductive organs of the LP group, arrow indicating the ovary; C, Reproductive organs of SP group, arrow indicating the ovary; D, Ovarian weight; E, Ovarian coefficient; LP, long photoperiods; SP, short photoperiods. n = 7, Values are the means ± SD. *P < 0.05 or **P < 0.01 means significant difference.

Adult female striped hamsters approximately 8 months of age were selected as experimental subjects. Body weights were measured before and after photoperiod exposure, and changes in body weight were analyzed accordingly. The results are presented in Fig 1(A). No significant difference in body weight was observed between the two groups of female striped hamsters either before or after photoperiod treatment (*P* > 0.05). After exposure to different photoperiods, the reproductive organs of female striped hamsters are presented in Fig 1B and 1C), including the uterus, fallopian tubes, ovaries (indicated by black arrows), and the adipose tissue surrounding the ovaries. Following 8 weeks of long photoperiod (LP) exposure, the uterus of striped hamsters appeared thicker and the ovaries were larger, whereas under short photoperiod (SP), the uterus was thinner, and the ovaries were smaller.

Subsequently, the bilateral ovaries were weighed after the fallopian tubes and periovarian fat were removed, and the results are presented in Fig 1(D). As shown in Fig 1(D), the ovarian weight of striped hamsters in the LP group was significantly higher than that in the SP group (*P* < 0.01). Furthermore, the ovarian coefficient in the LP group was significantly greater than that in the SP group (*P* < 0.05), as illustrated in Fig 1(E).

### 3.2. Differences in ovarian morphology and structure of striped hamsters under different photoperiods

Follicle is the fundamental functional unit of the ovary. Paraffin sectioning combined with HE staining was employed to examine variations in the morphological structure and quantity of follicles within the ovaries of the striped hamsters under different photoperiods. The morphological structures of follicles vary significantly across different developmental stages. Primary follicles contain only a single layer of cuboidal granulosa cells. Secondary follicles are characterized by multiple layers of granulosa cells and the presence of a complete zona pellucida. Antral follicles possess a clearly defined follicular antrum along with multiple layers of granulosa cells. In luteinized follicles, HE staining appears relatively lighter, and the granulosa cells differentiate into larger luteinized granulosa cells.

**Fig 2:**
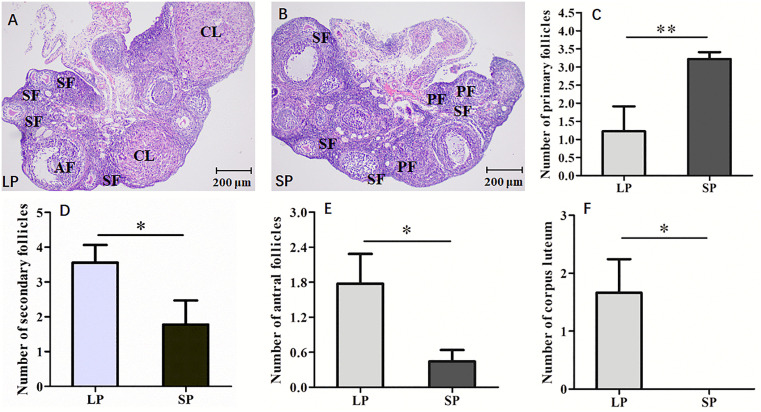
Differences in HE staining and the number of the follicles in ovary of the striped hamsters under different photoperiods. A, HE staining of ovary in LP; B, HE staining of ovary in SP; C, Number of primary follicles; D, Number of secondary follicles; E, Number of antral follicles; F, Number of corpus luteum; LP, long photoperiod; SP, short photoperiod; PF, primary follicles; SF, secondary follicles; AF, antral follicles; CL, corpus luteum. n = 3, Values are the means ± SD. *P < 0.05 or **P < 0.01 means significant difference.

The ovarian structures of the striped hamsters under long photoperiod (LP) and short photoperiod (SP) following HE staining are presented in Fig 2A and 2B, and distinct morphological differences were revealed between SP and LP groups. The ovarian sections from the LP group, as revealed by HE staining, exhibit a higher number of secondary follicles, along with clearly identifiable antral follicles and mature corpora lutea (Fig 2A). As illustrated in Fig 2B, ovaries from the SP group exhibited a significantly higher number of primary and secondary follicles, the lowest number of antral follicles, and a complete absence of mature corpora lutea. Further statistical analysis revealed that individuals in the LP group had a significantly lower number of primary follicles compared to those in the SP group (Fig 2C, *P* < 0.01). In contrast, both secondary and antral follicles were significantly more numerous in the LP group than in the SP group (Fig 2D and 2E, *P* < 0.05). In addition, no corpus luteum was observed in the striped hamsters from the SP group, whereas those from the LP group exhibited a significantly greater number of corpus luteum (Fig 2F, *P* < 0.05). The above results further demonstrate that photoperiod regulates the follicle development in the striped hamster. Exposure to a long photoperiod promotes the progression of primary follicles into secondary and antral follicles, whereas a short photoperiod suppresses the development of secondary and antral follicles.

### 3.3. The differences in the number and thickness of granulosa cell layers of follicles under different photoperiods

**Fig 3:**
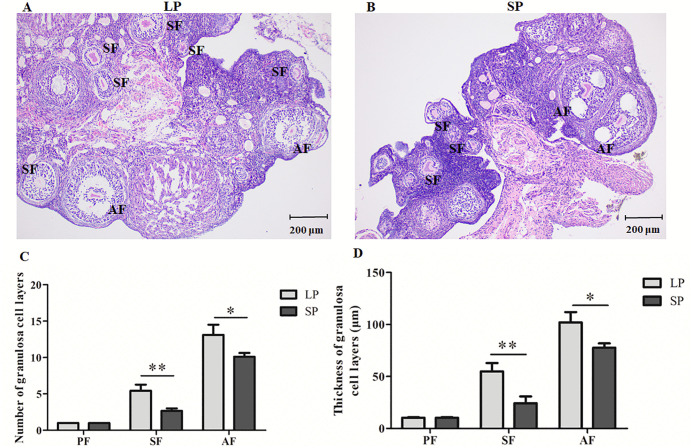
Variations in the number and thickness of follicle granulosa cell layers under different photoperiods. A, HE staining in LP; B, HE staining in SP; C, Number of follicle granulosa cell layers; D, Thickness of follicle granulosa cell layers; PF, primary follicle; SF, secondary follicle; AF, antral follicle; LP, long photoperiods; SP, short photoperiods; Bars, 200 µm. n = 3, Values are the means ± SD. *P < 0.05 or **P < 0.01 means significant difference.

During follicle development, the granulosa cell layer undergoes significant morphological and structural transformation. For example, as a secondary follicle progresses into an antral follicle, the granulosa cells surrounding the oocyte differentiate and organize to form a distinct follicle cavity. Therefore, dynamic alterations in the granulosa cell layer serve as a key indicator for assessing the developmental stages of follicles. As shown in Fig 3A, in LP group, the number of granulosa cell layers in secondary follicles typically ranges from 4 to 7, while that in antral follicles ranges from 12 to 14. However, as shown in Fig 3B, in SP group, most secondary follicles exhibited 2–3 layers of granulosa cells, with only a small minority displaying more than 3 layers. In the same SP group, the number of granulosa cell layers in antral follicles typically ranged from 9 to 10, and only a few antral follicles contained more than 10 layers of granulosa cells. Further statistical analysis revealed that the number of granulosa cell layers in both secondary follicles and antral follicles was significantly higher in LP group compared to SP group (Fig 3C).

In addition, the thickness of the granulosa cell layer in secondary follicles was 48–64 μm in the LP group and 19–32 μm in the SP group, while that in antral follicles was 92–113 μm in the LP group and 73–82 μm in the SP group. Consistent with the results of the granulosa cell layer number, the thickness of both secondary and antral follicle granulosa cell layers was significantly greater in the LP group than in the SP group (Fig 3D, *P* < 0.05).In both the LP and SP groups, primary follicles were surrounded by a single layer of granulosa cells, with the thickness of the granulosa cell layer ranging from 9 to 11 μm. No significant difference was observed between the two groups (*P* > 0.05).

The above results suggest that photoperiod may regulate follicle development in striped hamsters by influencing granulosa cell proliferation. Long photoperiod (LP) may stimulate granulosa cell proliferation, thereby promoting the development of secondary and antral follicles in striped hamsters; short photoperiod (SP) may partially inhibit granulosa cell proliferation, resulting in impaired development of secondary and antral follicles in striped hamsters.

### 3.4. The differences in hormone concentrations under different photoperiods and their correlation with the number of follicles

**Fig 4:**
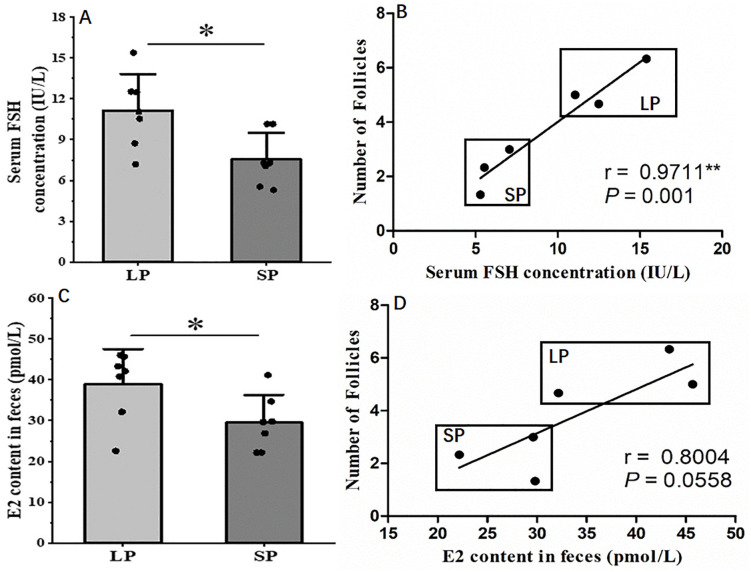
Differences of hormone concentration from different photoperiods and Correlation between the hormone concentration and the number of follicles. A, Serum FSH concentration (n = 7); B, Correlation between the serum FSH concentration and the number of follicles (n = 6); C, Fecal E2 content (n = 7); D, Correlation between the E2 concentration and the number of follicles (n = 6). LP, long photoperiod; SP, short photoperiod. Values are the means ± SD. r, correlation coefficient. *P < 0.05 means significant difference.

Follicle development is regulated by gonadotropins, among which FSH and E2 may serve as key regulatory factors. This study quantified the serum concentration of FSH and the feces content of E2 in striped hamsters, and analyzed the correlation between hormone concentrations and the number of follicles. The serum FSH concentration and the feces E2 content of the striped hamsters from LP group were significantly higher than those from SP group (Fig 4A and 4C, *P* < 0.05). The correlation analysis between hormone concentrations and the total number of secondary and antral follicles revealed that a significant positive association exists between FSH concentration and follicle count (Fig 4B, r = 0.9429, *P* = 0.0167), while no significant correlation was observed between E2 concentration and follicle count (Fig 4D, r = 0.7714, *P* = 0.1028). This result indicates that the FSH signaling pathway may mediate the effect of photoperiod on the development of secondary follicles and antral follicles in the striped hamsters. Long photoperiods may promote the synthesis and secretion of FSH in female striped hamsters, thereby facilitating follicle development, whereas short photoperiods may suppress FSH production and secretion, resulting in impaired follicle development. The synthesis and secretion of E2 are also regulated by the photoperiod, but the effect of E2-mediated photoperiod regulation on follicle development is not significant.

### 3.5. The differences in the expression levels of CCND1 and CCND2 at mRNA levels under different photoperiods and their correlation with the number of follicles

**Fig 5:**
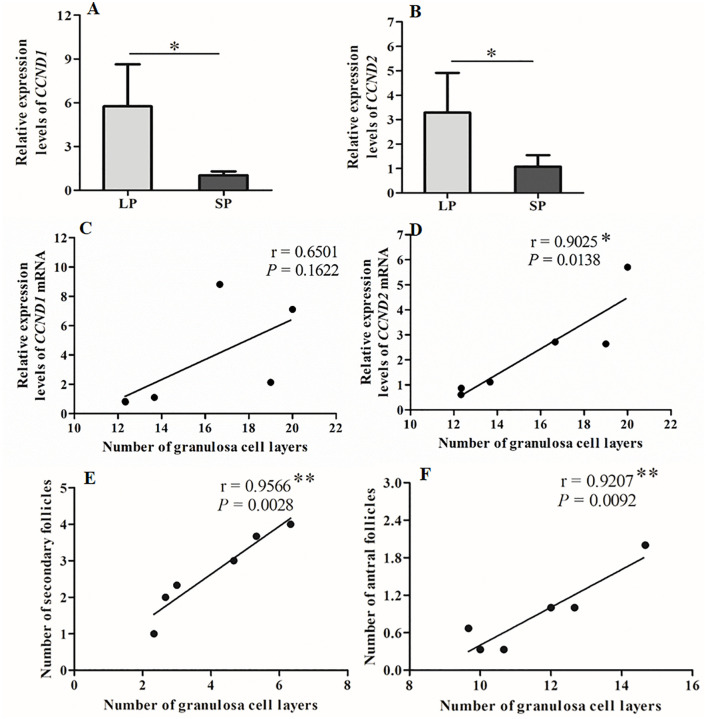
Differences in the expression levels of *CCND1* and *CCND2* mRNA under different photoperiods and their correlation with the number of follicle granulosa cell layers and follicles. A, *CCND1* mRNA; B, *CCND2* mRNA; C, Correlation between the *CCND1* mRNA and the number of granulosa cell layers; D, Correlation between the *CCND2* mRNA and the number of granulosa cell layers; E, Correlation between the number of granulosa cell layers and the number of secondary follicles; F, Correlation between the number of granulosa cell layers and the number of antral follicles; r, correlation coefficient; LP, long photoperiod; SP, short photoperiod. n = 6, Values are the means ± SD. *P < 0.05 and **P < 0.05 means significant correlation.

The relative mRNA of *CCND1* and *CCND2*, which are key cell proliferation regulatory factors, were detected in the ovaries of female striped hamsters under different photoperiods using real-time fluorescence quantitative PCR. The results demonstrate that the relative mRNA levels of *CCND1* and *CCND2* were significantly higher in the long photoperiod (LP) group compared to the short photoperiod (SP) group (Fig 5A, 5B, *P* < 0.05).The correlation between the relative mRNA levels of *CCND1* and *CCND2* and the number of granulosa cell layers in ovarian follicles is illustrated in Fig 5C and 5D. There was no significant correlation between the expression level of *CCND1* mRNA and the number of granulosa cell layers in the follicle (r = 0.6501, *P* = 0.1622). Nevertheless, the expression levels of *CCND2* mRNA showed significant positive correlations with the number of granulosa cell layers in follicles (r = 0.9025, *P* = 0.0138).The correlation analysis between the number of granulosa cell layers and the number of secondary follicles as well as antral follicles is presented in Fig 5E and 5F. Highly significant positive correlations were observed between the number of granulosa cell layers and both the number of secondary follicles (Fig 5E, r = 0.9566, *P* = 0.0028) and the number of antral follicles (Fig 5F, r = 0.9207, *P* = 0.0092).The above results suggest that photoperiod may regulate the expression of granulosa cell proliferation-related factors *CCND1* and *CCND2*, while *CCND2* mainly in turn influences granulosa cell proliferation within follicles and modulates follicle development in female striped hamsters. Therefore, the expression levels of *CCND2* can serve as potential biomarkers for detecting or regulating follicle development.

### 3.6. The variations in the expression of *FOXO1*, *FOXL2* and *NR5A2* at both mRNA and protein levels under different photoperiods

**Fig 6:**
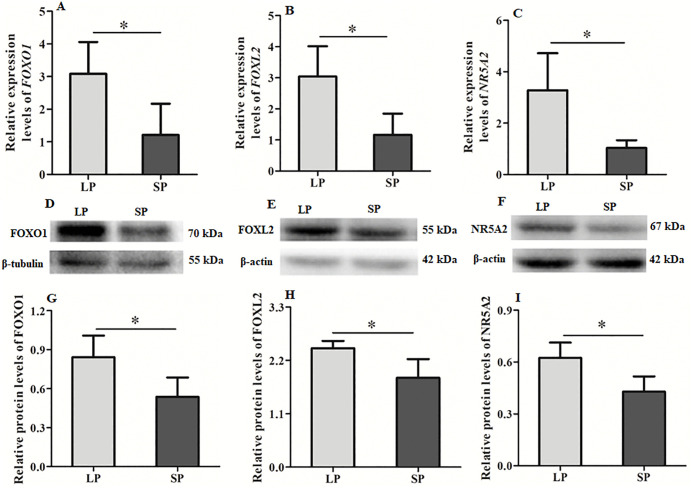
Differences in the mRNA and protein expression levels of *FOXO1*, *FOXL2* and *NR5A2* in the ovaries of striped hamsters under different photoperiods. A, *FOXO1* mRNA; B, *FOXL2* mRNA; C, *NR5A2* mRNA; D, Immunoblotting for *FOXO1* and β-actin; E, Immunoblotting *FOXL2* and β-actin; F, Immunoblotting for *NR5A2* and β-actin; G, *FOXO1* protein; H, *FOXL2* protein; I, *NR5A2* protein; LP, long photoperiod; SP, short photoperiod. n = 4, Values are the means ± SD. *P < 0.05 means significant difference.

The mRNA and protein expression levels of *FOXO1*, *FOXL2*, and *NR5A2*—key regulators involved in follicle development—in the ovaries of female striped hamsters were analyzed under different photoperiods using quantitative fluorescence PCR and Western blotting. The Western blot results of the follicle development-related factors FOXO1, FOXL2 and NR5A2 under different photoperiods are shown in Fig 6D, 6E and 6F. The expression levels of *FOXO1*, *FOXL2*, and *NR5A2* at both mRNA and protein levels in LP group were significantly higher than those in SP group (Fig 6). These findings suggest that the expression of these factors is regulated by photoperiod, thereby influencing follicle development.

### 3.7. The correlation between the expression levels of follicle development-related factors in the ovaries of female striped hamsters

FSH serves as the primary hormone responsible for regulating follicle development. A significant positive correlation was found between serum FSH concentration and both the mRNA and protein expression levels of the transcription factors FOXO1 and FOXL2 (Table 2). This result indicates that the expression of transcription factors FOXL2 and FOXO1 in the ovary of striped hamsters may be both regulated by the FSH signaling pathway. No significant correlation was observed between the mRNA expression levels of FOXO1 and FOXL2 and those of NR5A2; however, a significant positive correlation was detected at the protein expression level (Table 2). The expression levels of NR5A2 showed highly significant positive correlations with those of CCND2 at mRNA levels (Table 2). The analysis results indicate that FOXO1, FOXL2 and NR5A2 may play significant roles in the FSH response to photoperiodic regulation of follicle development in the striped hamster. The correlations between FOXO1 and NR5A2, as well as between FOXL2 and NR5A2, are stronger (r = 0.8690, *P* = 0.0051; r = 0.9158, *P* = 0.0014) than those between FSH and FOXO1 (r = 0.7924, *P* = 0.0190) and between FSH and FOXL2 (r = 0.7113, *P* = 0.0479), indicating that NR5A2 may serve as a more effective target for regulating reproductive activity in the striped hamster (*Cricetulus barabensis*).

**Table 2:** Correlation between factors associated with follicle development.

Factors related to follicle development	mRNA level	protein level
FSH/FOXO1	r = 0.8231*, *P* = 0.0121	r = 0.7924*, *P* = 0.0190
FSH/FOXL2	r = 0.7755*, *P* = 0.0237	r = 0.7113*, *P* = 0.0479
FOXO1/ NR5A2	r = 0.5640, *P* = 0.1453	r = 0.8690**, *P* = 0.0051
FOXL2/ NR5A2	r = 0.4768, *P* = 0.2326	r = 0.9158**, *P* = 0.0014
NR5A2/ CCND2	r = 0.9112**, *P* = 0.0016	

Note: n = 8, *P < 0.05 or **P < 0.01 indicates a significant correlation.

## 4. Discussion

This study primarily investigated the impact of photoperiod on follicle development, as well as the effects of CCND1 and CCND2 on granulosa cell proliferation within the ovarian follicles. Furthermore, it explored the involvement of the transcription factors FOXO1, FOXL2 and NR5A2 in the pathway through which FSH mediates the effects of photoperiod on follicle development in the striped hamster.

### 4.1. The effects of photoperiod on body weight, ovarian weight and ovarian coefficient of the striped hamster

Seasonal breeding animals perceive external environmental cues, which regulate hormonal secretion and gonadal development, thereby influencing their reproductive activities [50]. Photoperiod serves as the most reliable environmental cue for seasonal breeding animals to detect changes in their external environment and subsequently regulate their reproductive activities. The striped hamster exhibits typical seasonal breeding characteristics. In this study, adult female striped hamsters were treated with different photoperiods. No significant difference were detected in body weight between the LP and the SP groups of the striped hamsters. However, the ovarian weight and ovarian coefficient of the LP group were significantly higher than those of the SP group, revealing that photoperiod affects the structure of the ovaries in the striped hamsters. A study demonstrates that prolonged light exposure leads to an increase in body weight and a significant elevation in both ovarian weight and ovarian coefficient in *Phodopus sungorus* [51]. Sriparna *et al*. demonstrated that golden hamsters exposed to long photoperiods exhibited a reduction in body weight, accompanied by increases in ovarian weight and ovarian coefficient [52]. These findings indicate that the effects of photoperiod on body weight differ across animal species, whereas its influence on ovarian weight remains consistent. Specifically, long photoperiods promote ovarian development, while short photoperiods suppress it.

The ovary, recognized as the most critical reproductive organ in female animals, is responsible for synthesizing and secreting sex hormones as well as producing mature oocytes. Research indicates that impaired ovarian development in mice can diminish their litter size, thereby reducing reproductive capacity [53].Therefore, ovarian development is closely correlated with the intensity of reproductive activity in female animals. Extended photoperiods promote ovarian development in female striped hamsters, thereby facilitating reproductive activity, whereas shortened photoperiods induce ovarian atrophy and degeneration, which may result in a partial suppression of reproductive function.

### 4.2. The effect of photoperiod on granulosa cell proliferation and follicle development in striped hamsters

Follicles represent the basic functional units of the ovary, and their growth and development are strongly associated with the reproductive capacity of female animals. Evidence suggests that enhanced follicle development can increase litter size in mice and rabbits, consequently improving their reproductive performance [54,55]. Based on the structural characteristics of follicles at different developmental stages, they are categorized into primordial follicles, primary follicles, secondary follicles, antral follicles and mature follicles. Following ovulation, mature follicles transform into the corpus luteum. In this study, HE staining of ovarian sections from the female striped hamsters exposed to different photoperiods revealed that all developmental stage follicles were present in the long photoperiod (LP) group. Among these, secondary follicles were most abundant, followed by antral follicles and corpora lutea, while primary follicles were the least numerous. In contrast, the short photoperiod (SP) group exhibited a high number of primary follicles, fewer secondary and antral follicles, and no mature corpora lutea were detected. These observations align with the findings reported by Kabithe *et al*. in Siberian hamsters [56]. The LP group exhibited a significantly lower number of primary follicles compared to the SP group, whereas the number of secondary follicles was significantly higher in the LP group. These findings are consistent with those of Sriparna *et al*., who studied golden hamsters under short photoperiods [52]. The aforementioned results demonstrate that long photoperiod facilitates the transition of primary follicles into secondary follicles in female striped hamsters, whereas short photoperiods may suppress the development of secondary follicles. The LP group exhibited a significantly greater number of antral follicles compared to the SP group, a finding that aligns with the results reported by Leon *et al*. in adult Siberian hamsters [57]. These observations suggest that LP may facilitate the progression of secondary follicles into antral follicles and enhance the possibility of antral follicles maturing to the stage of ovulation. The LP group showed a significantly higher number of corpora lutea compared to the SP group. Moreover, no mature corpora lutea were detected in the ovaries of the SP group, a finding that aligns with the results reported by Salomon *et al*. regarding the ovaries of adult Siberian hamsters under SP conditions [58]. The corpus luteum is an adenoid structure that forms following the ovulation of a mature follicle. An increased number of corpora lutea further supports the promoting effect of long photoperiod (LP) on follicle development, indicating that LP conditions facilitate oocyte maturation and ovulation, thereby enhancing reproductive capacity.

The follicle is primarily composed of oocytes, granulosa cells, and theca cells. As a critical component of the follicle, granulosa cells play a central role in regulating the secretion and synthesis of factors associated with oocyte development, thereby contributing to the overall development of the follicle. Studies have shown that follicle development is heavily reliant on the proliferation of granulosa cells [59]. Throughout the course of follicle development, a concurrent increase in both the number of granulosa cell layers and their thickness surrounding the oocyte has been observed [18]. As the number of granulosa cells increases, gaps emerge between these cells, which ultimately coalesce to form a relatively large follicular cavity [60]. The granulosa cells of primary follicles are only one layer with a thickness ranging from 9 to 11 μm. No statistically significant difference in granulosa cell thickness is observed between primary follicles in the long photoperiod (LP) and short photoperiod (SP) groups. The number and thickness of granulosa cell layers in secondary and antral follicles are significantly greater in LP group compared to SP group, indicating that LP promotes granulosa cell proliferation and thereby enhances follicle development. This result aligns with the findings of Zheng *et al*., demonstrating that the development of porcine follicles is largely dependent on granulosa cell proliferation [61]. Based on the results, photoperiod regulates granulosa cell proliferation within follicles, thereby influencing the process of follicle development. LP stimulates granulosa cell proliferation, promoting the development of secondary and antral follicles, which subsequently facilitates oocyte maturation and ovulation. LP enhances the reproductive capacity in animals, whereas SP exerts the opposite effect.

### 4.3. The impact of photoperiod on the synthesis of FSH, CCND1 and CCND2, and its correlation with the number of follicles

FSH plays a crucial role in follicle development. Studies have demonstrated that LP exposure increases serum FSH concentration, upregulates the expression of genes associated with granulosa cell proliferation and estradiol (E2) synthesis [52,62], thereby promoting the development of secondary and antral follicles [62]. This study demonstrated that serum FSH concentration and fecal estradiol (E2) levels in the LP group were significantly higher than those in the SP group, a finding that aligns with previous research findings. The above results suggest that photoperiod modulates the synthesis of FSH in the hypothalamic-pituitary-ovarian (HPO) axis and estradiol (E2) in the ovaries of striped hamsters.

Correlation analysis revealed that FSH levels exhibited a significant positive correlation with the number of secondary and antral follicles, whereas no significant association were detected between E2 levels and the number of follicles. These findings align with those of Chen *et al*., who demonstrated that FSH can stimulate granulosa cell proliferation in rats, thereby promoting follicle development [63]. Therefore, it is hypothesized that FSH plays a more prominent role in mediating photoperiod to regulate follicle development.

Cyclin D1 (CCND1) and Cyclin D2 (CCND2) are key regulators of cell proliferation in various cell types, including glioma cells [64], placental trophoblast cells [65], and granulosa cells [66]. CCND1 and CCND2 facilitate the progression of cells from the G1 phase to the S phase through the activation of CDK4 or CDK6, thereby promoting sustained cell proliferation [27]. Importantly, the proliferative potential of granulosa cells serves as a key determinant in follicle development. CCND1 and CCND2 serve as key regulatory factors controlling granulosa cell proliferation in mice [66]. The follicle development largely depends on the proliferation of granulosa cells in porcine [61]. This study revealed that the expression levels of *CCND1* and *CCND2* mRNA in the ovaries of striped hamsters under LP conditions were significantly higher compared to those observed under SP conditions. Moreover, the *CCND2* mRNA was significantly positively correlated with the number of granulosa cell layers in the follicles. These results indicate that photoperiod regulates the expression of *CCND1* and *CCND2* in the ovaries of striped hamsters, and CCND2, in turn, control the proliferation of granulosa cells within the follicles, thereby influencing follicle development. The findings of this study align with those reported by Liu *et al*., further supporting the conclusion that CCND2 serve as indispensable regulatory factors in the proliferation of mammalian granulosa cells and the development of ovarian follicles [28,29].

### 4.4. The effect of photoperiod on the expression of transcription factors related to follicle development

FSH, by binding to its receptor FSHR, plays a pivotal role in regulating follicle development and the proliferation of granulosa cells [67]. Coelho *et al*.‘s findings indicate that the relative expression level of *FSHR* mRNA is significantly elevated under long photoperiods [68]. Cui *et al*. demonstrated that extending the duration of light exposure to 14–16 hours leads to a significant increase in serum FSH concentration in laying ducks, accompanied by a corresponding upregulation of *FSHR* mRNA and protein expression levels in the ovaries [69,70]. This study demonstrated that serum FSH concentration in the LP group were significantly higher compared to those in the SP group. These findings demonstrate that LP enhances the increasing of the serum FSH concentration, thereby stimulating granulosa cell proliferation and promoting the further development of secondary and antral follicles. In contrast, SP suppresses the serum FSH concentration, which hinders the transmission of FSH signals into granulosa cells, leading to reduced cell proliferation and impaired follicle development in the striped hamster.

Upon binding of FSH to FSHR, the signal is transmitted from the extracellular environment into the granulosa cells. This signal then activates transcription factors through a complex intracellular signaling network and is further relayed to the nucleus. Ultimately, the expression of downstream target genes of these transcription factors is modulated, thereby influencing the follicle development in animals [71]. FOXO1, FOXL2, and NR5A2 are key transcription factors involved in the regulation of granulosa cell proliferation and play essential roles in follicle development across animal species [37,72]. The results of this study demonstrate that the expression levels of FOXO1, FOXL2, and NR5A2 at both mRNA and protein levels in the ovaries of the LP group of striped hamsters were significantly higher than those in the SP group. These findings reveal that the transcription factors FOXO1, FOXL2 and NR5A2 play a crucial role in photoperiod-regulated follicle development in striped hamsters.

The transcription factor FOXO1 plays a critical role in the regulation of follicle development [40]. Knockout of the FOXO1 gene results in impaired granulosa cell proliferation, suppressed follicle development, and ovarian atrophy and degeneration in mice [42]. Upregulation of FOXO1 expression promotes granulosa cell proliferation and facilitates the development of secondary follicles in rats [73]. Emerging evidence also indicates that FOXO1 knockdown enhances granulosa cell proliferation in cattle and sheep, thereby promoting follicle development [74]. This suggests that the regulatory effects of FOXO1 on granulosa cell proliferation and follicle development differ across species, which may partially explain why reproductive activities in different species occur under varying photoperiods. The results of this study demonstrate that LP enhances FOXO1 expression in the ovaries of striped hamsters at both mRNA and protein levels, whereas SP reduces FOXO1 expression. Based on the findings, it is hypothesized that LP enhances FOXO1 expression in the ovaries of striped hamsters, which in turn promotes granulosa cell proliferation and follicle development.

The expression of FOXL2 in animal ovaries is regulated by the photoperiod, and emerging evidence suggests that FOXL2 plays a critical role in the development of ovarian follicles. Wang *et al*.‘s study on the ovaries of silver pomfret revealed that the expression levels of *FOXL2* at both the mRNA and protein levels were significantly higher under LP conditions than those under SP conditions [75]. Lee *et al*. observed that the ovaries of Siberian hamsters underwent atrophy under SP conditions, and the degree of FOXL2 immunostaining in the SP group was significantly reduced compared to that in the LP group [76]. In this study, the expression level of *FOXL2* in the ovaries of striped hamsters increased as the photoperiod was extended. LP upregulated the expression of the transcription factor *FOXL2*, whereas SP led to its downregulation. Emerging evidence has demonstrated that the knockdown of *FOXL2* expression in chicken granulosa cells promotes granulosa cell proliferation [77]. In rats, upregulation of *FOXL2* expression suppresses granulosa cell proliferation and triggers granulosa cell apoptosis, thereby negatively affecting follicle development [78]. Therefore, it is hypothesized that FOXL2 plays a crucial role in the photoperiod-mediated regulation of follicle development; however, its regulatory effect exhibits species-dependent. In striped hamsters, long photoperiod (LP) enhances *FOXL2* expression in the ovaries, and FOXL2 subsequently promotes granulosa cell proliferation and follicle development, consistent with findings in Siberian hamsters [76].

As a key transcription factor involved in the regulation of follicle development, NR5A2 is specifically localized in the granulosa cells of the follicle. Nearly all of its downstream target genes are implicated in the follicle development, including those encoding cyclins and ovulation-related factors [79]. Emerging evidence indicates that upregulation of NR5A2 expression in granulosa cells across humans, mice, Siberian hamsters, and Hu sheep promotes granulosa cell proliferation [80,81]. Recent studies have demonstrated that upregulation of NR5A2 expression in bovine granulosa cells suppresses granulosa cell proliferation, potentially triggering apoptosis and ultimately leading to follicle atresia in cattle [82]. Therefore, it can be inferred that the effect of NR5A2 on follicle development in animals exhibits interspecies variation. Leon *et al*. demonstrated that NR5A2 is among the genes exhibiting significant differential expression in the ovaries of Siberian hamsters under varying photoperiods. The results of this study demonstrate that LP enhance the expression of *NR5A2* in the ovaries of striped hamsters at both the mRNA and protein levels, whereas SP reduce *NR5A2* expression. These findings are consistent with those reported by Leon *et al*.. In summary, the expression of *FSHR* and the transcription factors *FOXO1*, *FOXL2* and *NR5A2* in animal ovaries is regulated by photoperiod; however, the effects of these transcription factors—FOXO1, FOXL2, and NR5A2—on follicle development exhibit species-specific characteristics. In this study, LP also upregulates *NR5A2* expression in the ovaries of striped hamsters, and NR5A2 further stimulates granulosa cell proliferation and follicle development, aligning with results reported in Siberian hamsters [57,80].

### 4.5. The potential pathway of follicle development regulated by FSH in response to photoperiod in striped hamsters

The synthesis and secretion of FSH are regulated by the photoperiod [52]. The binding of FSH to FSHR regulates granulosa cell proliferation and follicle development via a complex signaling network [83]. Elevated FSH concentration stimulates the synthesis of hyaluronic acid, thereby promoting the proliferation and expansion of the granulosa cell layer [84]. FSH binding to FSHR activates the PI3K/Akt signaling pathway in mouse granulosa cells, thereby inhibiting the activity of the serine/threonine kinase GSK3β and promoting the expression of the transcription factor OCT4, which contributes to granulosa cell proliferation and follicle development from preantral to antral stages [85]. Some studies have also demonstrated that FSH binding to FSHR stimulates the expression of *IGF-2* in human granulosa cells, activates the PI3K/Akt and ERK signaling pathways to upregulate *CYP19A1* expression, and promotes granulosa cell proliferation [75]. Further studies indicate that the binding of FSH to FSHR enhances *HIF1* expression, which subsequently upregulates the expression of *CCND2*, thereby promoting granulosa cell proliferation [86]. Therefore, it is hypothesized that granulosa cell proliferation is under the regulation of FSH; however, the specific regulatory network differ across species. Some studies have also demonstrated that FSH binding to its receptor FSHR initiates intracellular signaling, which affects the activity of FOX family transcription factors through the activation of PKA-dependent serine/threonine kinases in the PI3K/Akt signaling pathway, thereby regulating the expression of genes associated with granulosa cell proliferation and follicle development [44–46]. This study demonstrates that the expression levels of *FOXO1* and *FOXL2* at both the mRNA and protein levels exhibit a significant positive correlation with serum FSH concentration. These findings suggest that the expression of FOX family transcription factors in the ovaries of striped hamsters may be modulated by the FSH signaling pathway.

Studies have shown that the promoter region of NR5A2 contains a high density of binding sites for FOX transcription factors, suggesting that these factors may regulate *NR5A2* expression either directly or indirectly [34]. In this study, the protein expression levels of *FOXO1* and *FOXL2* were significantly positively correlated with those of *NR5A2*. These findings provide further evidence suggesting that FOXO1 and FOXL2 may function as transcription factors regulating the expression of *NR5A2* in the ovaries of striped hamsters.

Meinsohn *et al*. demonstrated that NR5A2 regulates the proliferation of mouse granulosa cells by regulating the expression of the cell cycle regulators *CCND1* and *CCND2*, thereby further proving NR5A2 as an essential regulator of granulosa cell proliferation [66]. In this study, the expression levels of *NR5A2* at both the mRNA and protein levels showed highly significant positive correlations with those of *CCND2* at corresponding molecular levels. These findings suggest that the transcription factor NR5A2 may regulate follicle development in striped hamsters by modulating the expression of the cell proliferation regulators CCND2. Andreu *et al*. also demonstrated that elevated serum FSH concentrations induce increased FSHR expression, accompanied by upregulation of NR5A2, thereby promoting the proliferation of both mouse and bovine granulosa cells [87,88]. These findings suggest that NR5A2 may serve as a key regulatory component in the FSH signaling pathway, contributing to the regulation of follicle development in striped hamsters.

**Fig 7:**
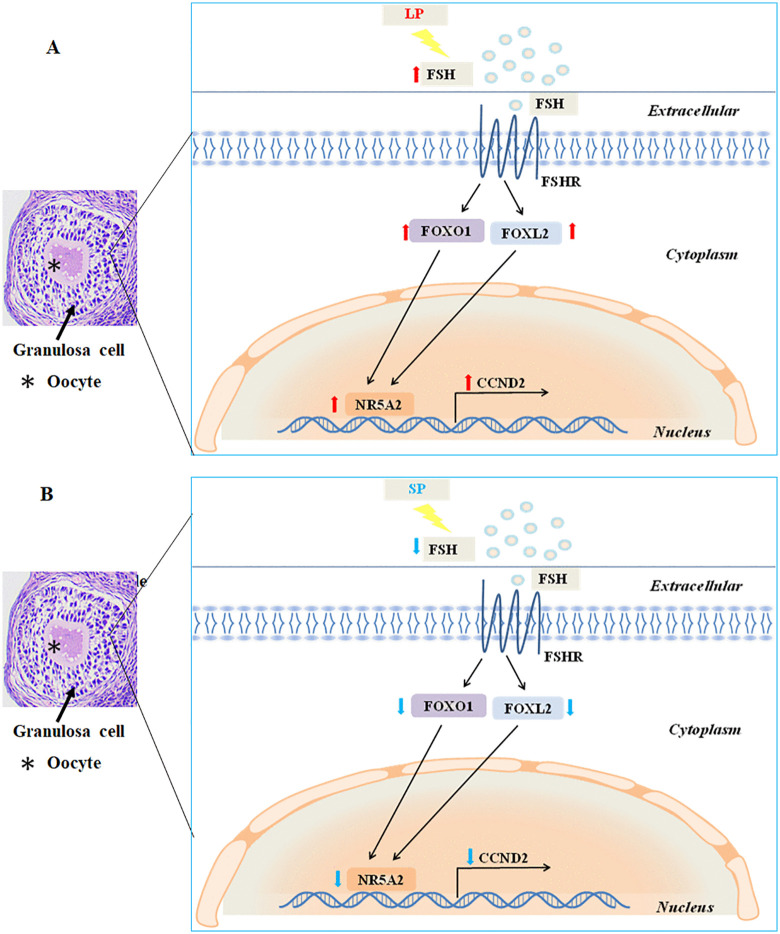
Molecular mechanism diagram of the photoperiod regulating follicle development in female striped hamsters.

The above results indicate that the expression of the transcription factor NR5A2 in the ovaries of striped hamsters is predominantly regulated by the FSH-FOX signaling pathway. The FSH-FOX-NR5A2 signaling pathway plays a crucial role in photoperiod-regulated follicle development in striped hamsters by modulating the expression of the cell proliferation regulators *CCND2*. Under LP, the serum FSH concentration in striped hamster increases. Upon FSH binding to FSHR, the expression of transcription factors *FOXO1* and *FOXL2* is upregulated, which subsequently enhances the expression of the transcription factor *NR5A2*, thereby promoting the expression of the cell proliferation regulators *CCND2* (Fig 7A). However, under SP, the serum FSH concentration decreases, thereby impairing FSH signal transduction. This leads to downregulation of the transcription factors *FOXL2* and *FOXO1*, which subsequently suppresses the expression of the transcription factor *NR5A2*, resulting in decreased expression of the cell proliferation regulators *CCND2* (Fig 7B). This result suggests that the FSH-FOX-NR5A2 signaling pathway may serve as a potential mechanism mediating photoperiod-regulated proliferation of granulosa cells by modulation the expression of the cell proliferation regulators *CCND2*, ultimately influencing follicle development in striped hamsters.

Future studies employing specific inhibitors—such as those targeting FSH signaling, FOXO1/FOXL2, or NR5A2—in an in vitro granulosa cell culture system will be necessary to establish direct causal relationships within this pathway.

## 5. Conclusions

In summary, this study exposed adult female striped hamsters to varying photoperiods to investigate the molecular mechanisms underlying photoperiod-regulated follicle development in these animals. Studies have shown that LP stimulates granulosa cell proliferation, thereby promoting follicle development in striped hamsters and supporting ovarian growth. In contrast, SP suppresses granulosa cell proliferation, hinder follicle development, and ultimately lead to ovarian atrophy and degeneration in these animals. During this process, the FSH-FOX-NR5A2-CCND2 signaling pathway exerts a crucial regulatory function. FSH binding to FSHR primarily modulates the expression of the transcription factor *NR5A2* through regulation of the transcription factors FOXO1 and FOXL2. Altered expression levels of *NR5A2* subsequently influence the expression of the cell proliferation regulators *CCND2*, thereby affecting granulosa cell proliferation and ultimately regulating follicle development in the striped hamster. This study demonstrates that the FSH-FOX-NR5A2-CCND2 signaling pathway mediates photoperiod-regulated granulosa cell proliferation in follicles based on strong correlative evidence, thereby influencing follicle development in the striped hamster. In this study, exposure to a long photoperiod (LP) upregulates the expression of *FOXO1*, *FOXL2*, and *NR5A2* in the ovaries, thereby promoting granulosa cell proliferation and follicle development. Among these genes, NR5A2 exhibits a stronger correlation with the observed effects compared to FOXO1 and FOXL2. Therefore, NR5A2 may serve as a more effective target for regulating reproductive activity in the striped hamster (*Cricetulus barabensis*).These findings suggest that it may represent one of the key molecular mechanisms underlying seasonal reproductive activities in small rodents inhabiting temperate regions.

## Supporting information

S1 File S1. Body weight-Ovarian Weight-Ovarian coefficient. S2. Reproductive organs. S3. HE Pictures. S4. Follicle Number and GC layers. S5. Hormones. S6. qPCR Results. S7. Supporting information for Western Blotting. S8. WB Results.
(ZIP)
