# Supplementary figures and images for: Potential FSH-mediated molecular pathway to regulate follicle development in striped hamsters (Cricetulus barabensis) supported by strong correlative evidence
Source: PLoS One. 2025 Dec 29;20(12):e0339880. doi: 10.1371/journal.pone.0339880 (PMC12747357; doi:10.1371/journal.pone.0339880)

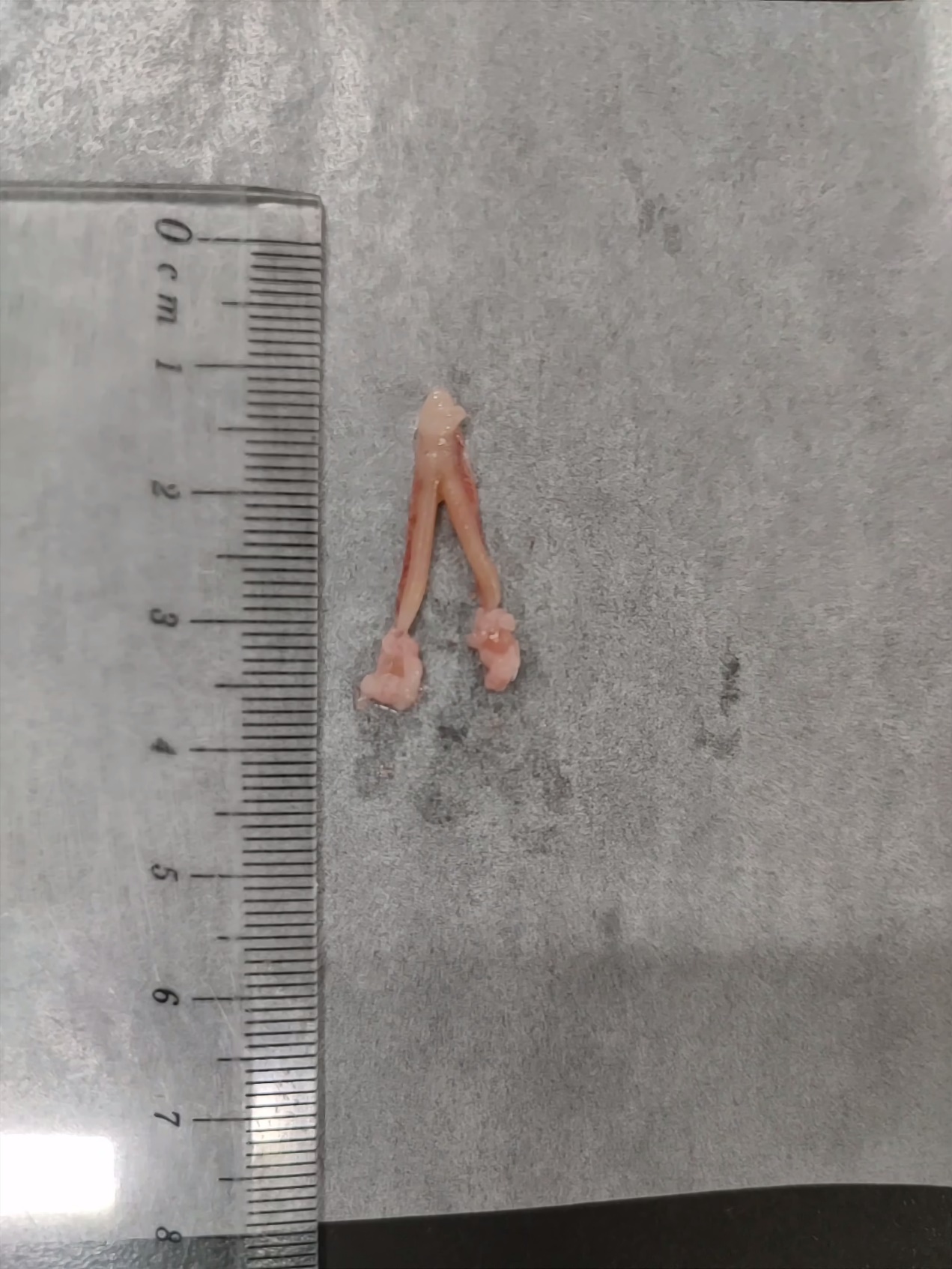


Reproductive organs on LP


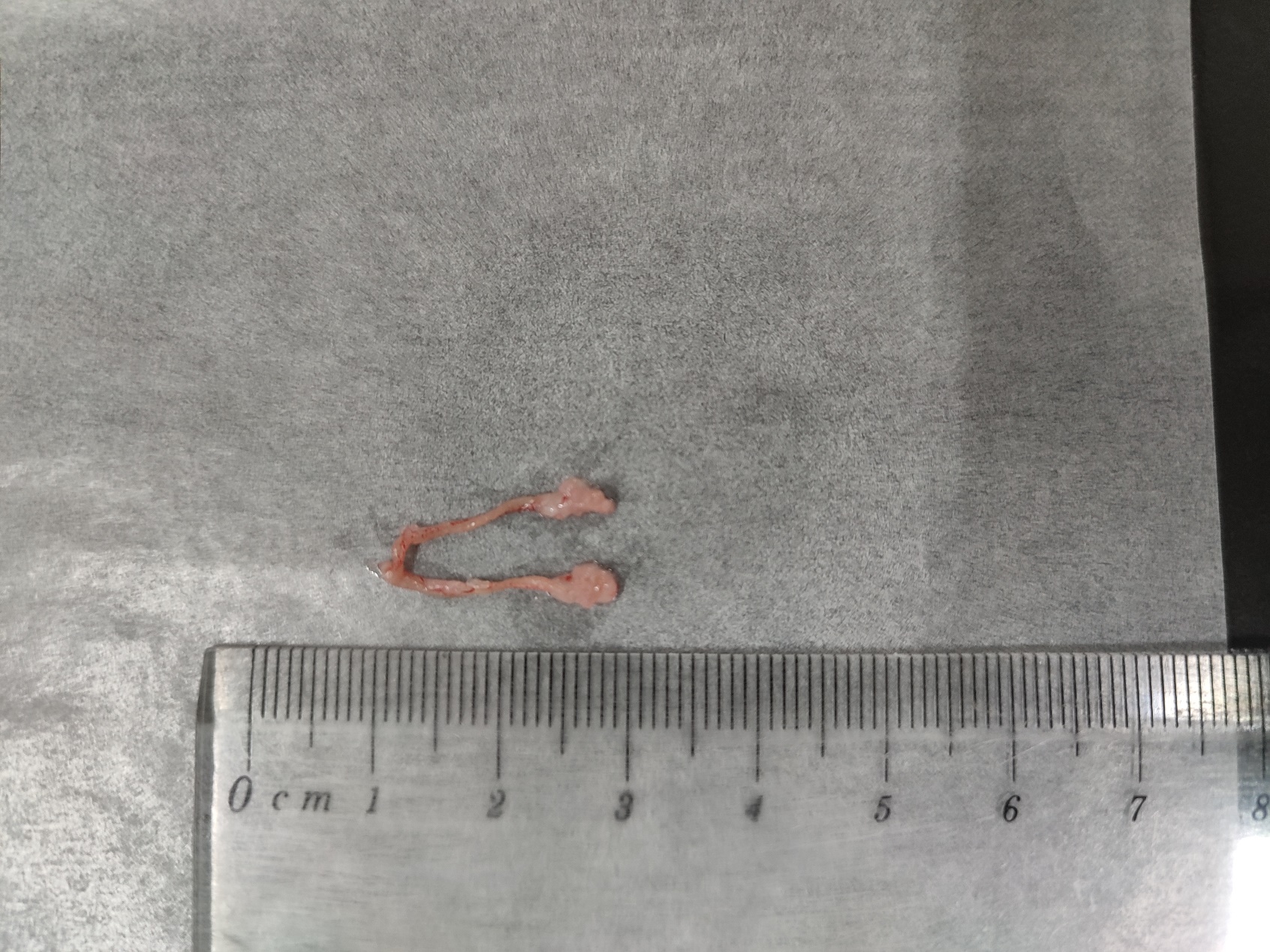


Reproductive organs on SP

Supplement: S1 File — S1. Body weight-Ovarian Weight-Ovarian coefficient. S2. Reproductive organs. S3. HE Pictures. S4. Follicle Number and GC layers. S5. Hormones. S6. qPCR Results. S7. Supporting information for Western Blotting. S8. WB Results. (ZIP) [file pone.0339880.s001.zip › Supporting Information for data/2. Reproductive organs.docx]

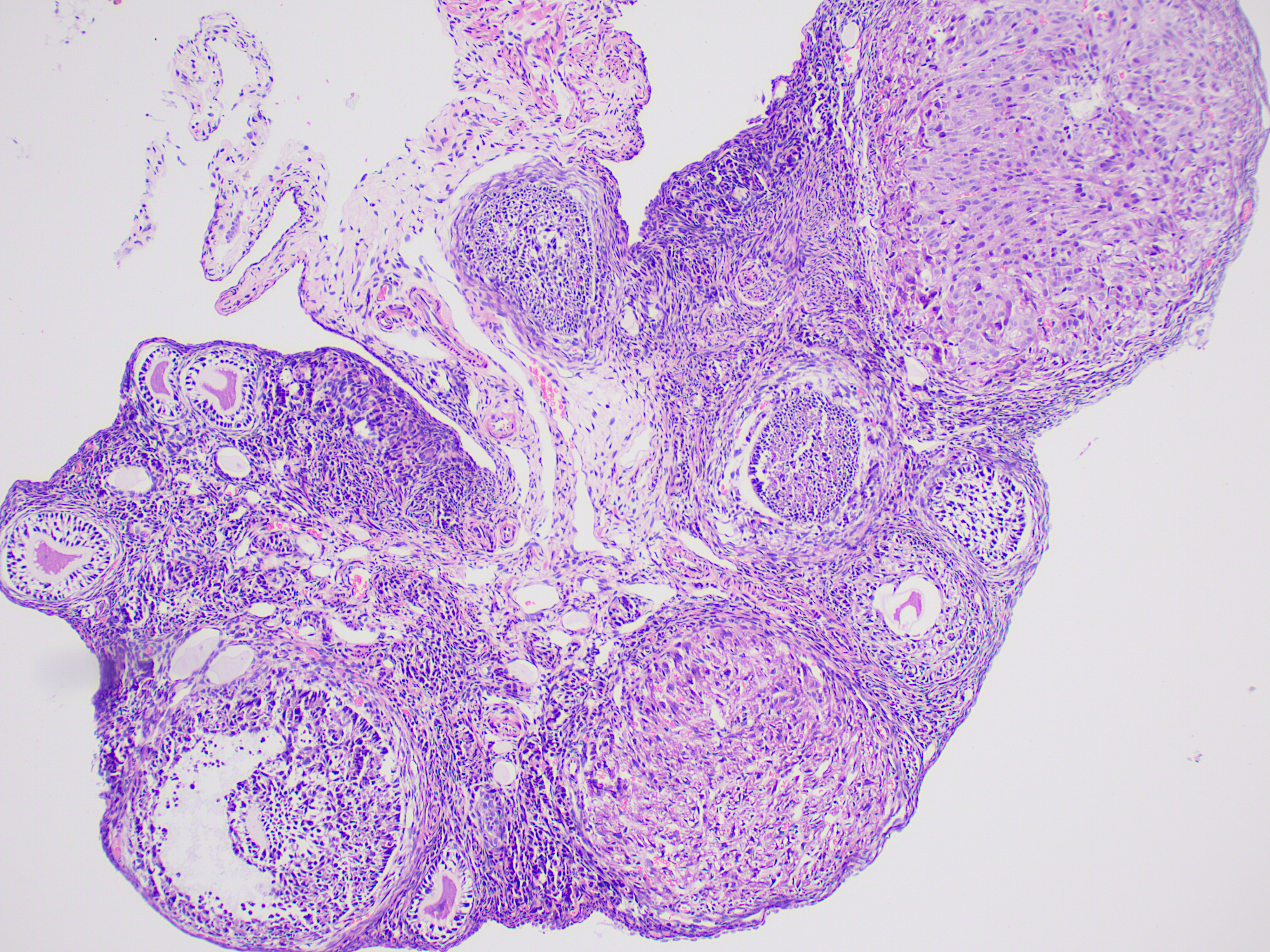


Figure 2 A


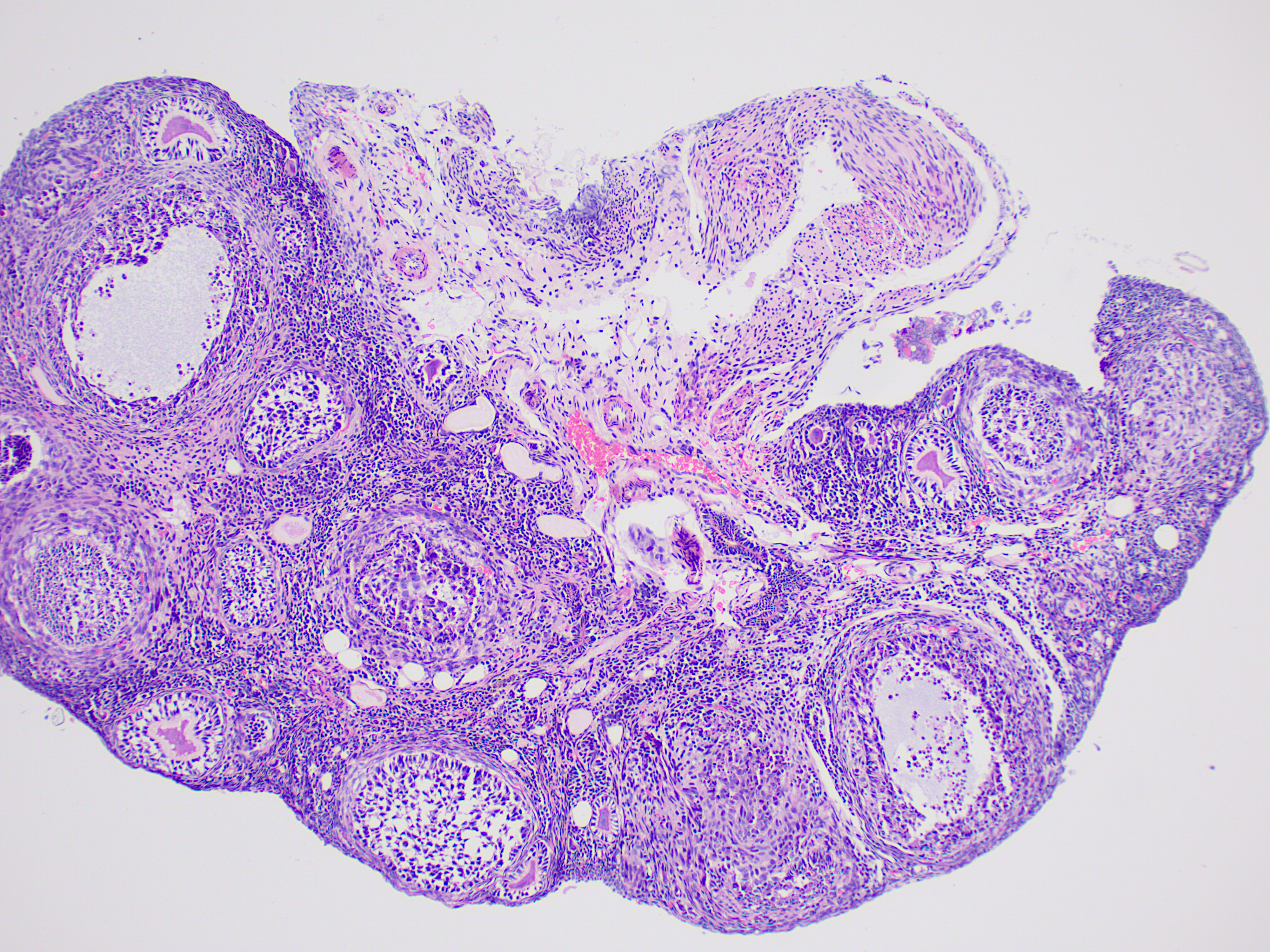


Figure 2 B


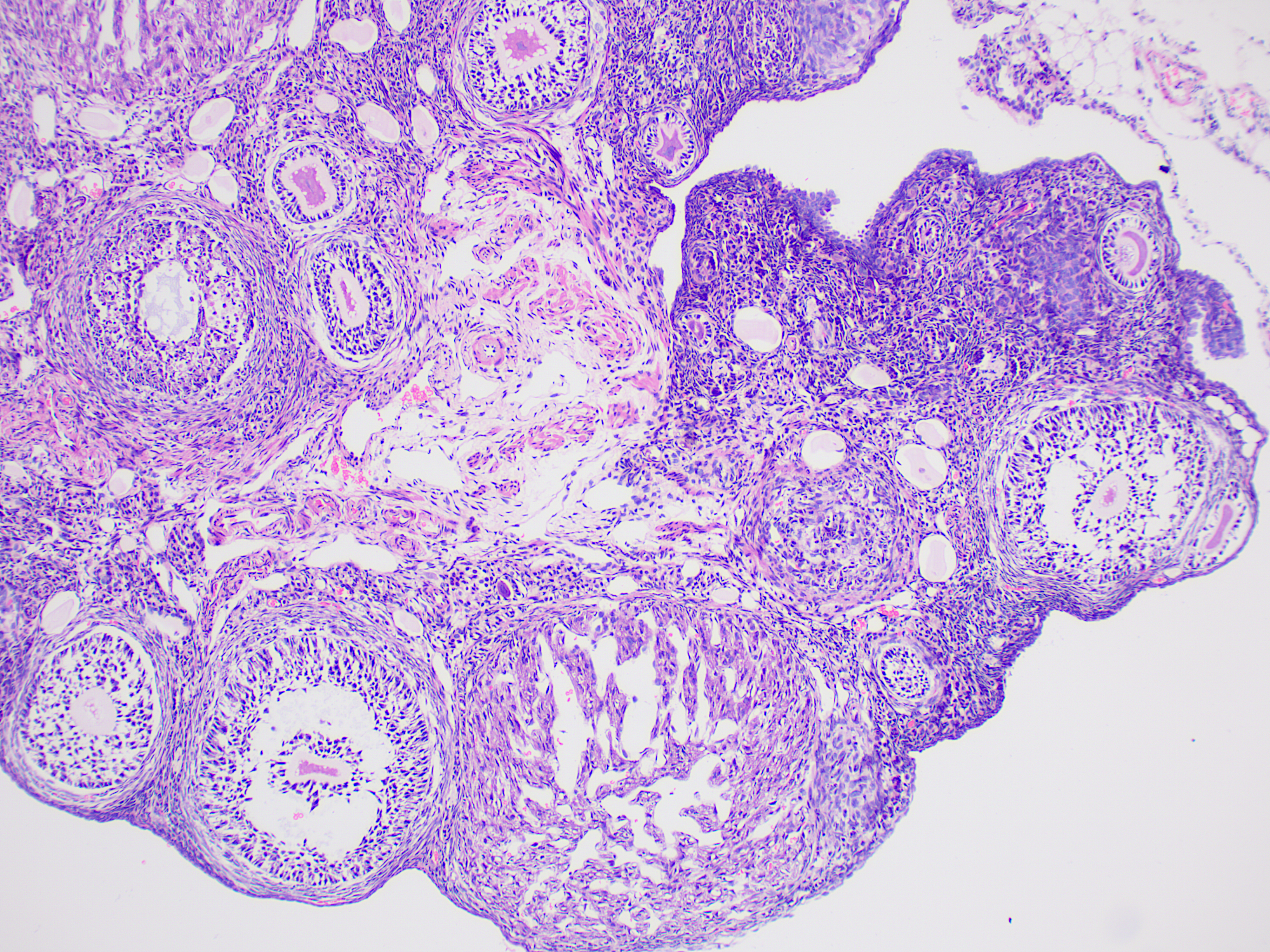


Figure 3 A


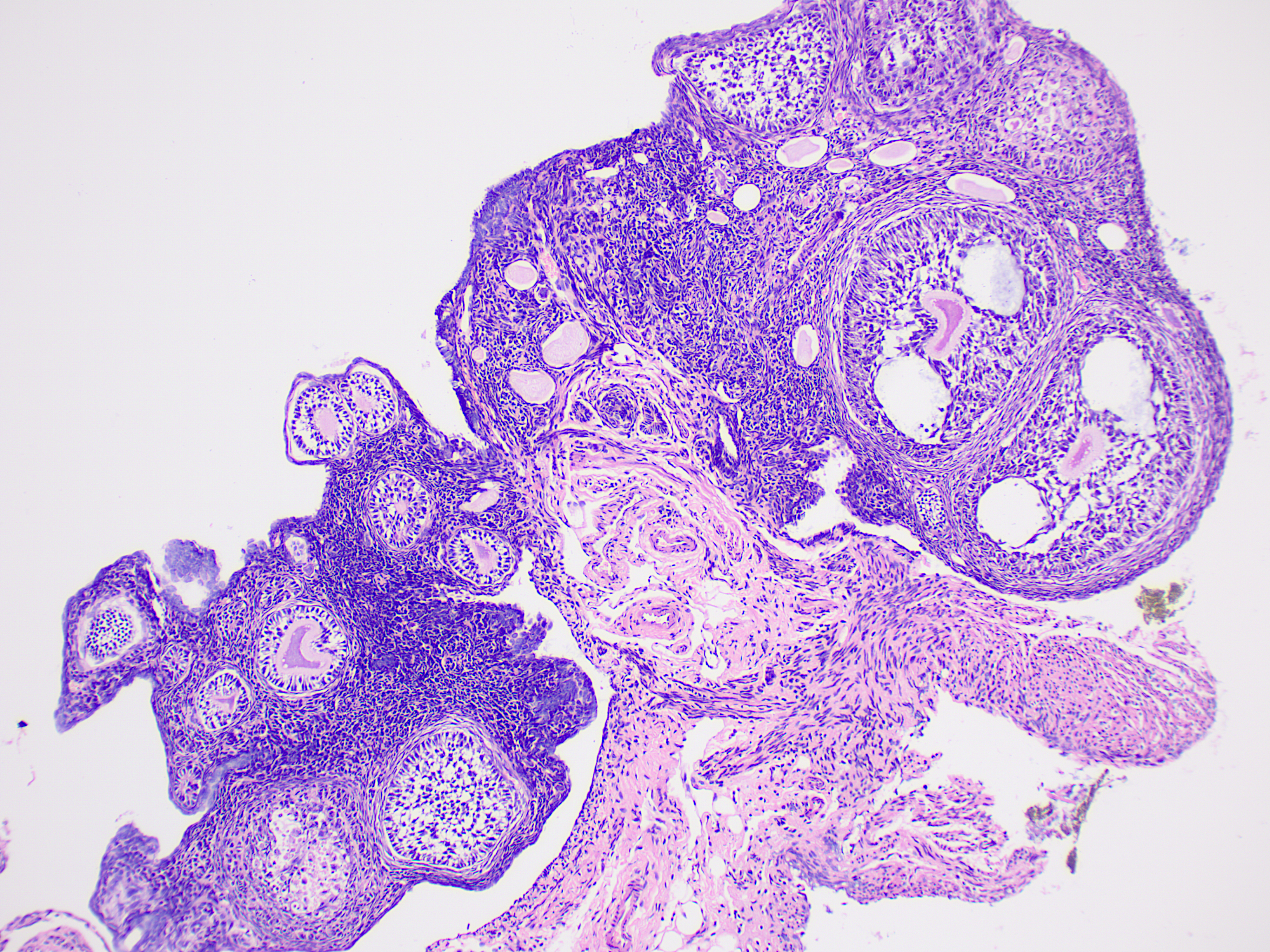


Figure 3 B

Supplement: S1 File — S1. Body weight-Ovarian Weight-Ovarian coefficient. S2. Reproductive organs. S3. HE Pictures. S4. Follicle Number and GC layers. S5. Hormones. S6. qPCR Results. S7. Supporting information for Western Blotting. S8. WB Results. (ZIP) [file pone.0339880.s001.zip › Supporting Information for data/3 HE Picture.docx]
